# Synthesis and evaluation of ^68^Ga-labeled dimeric cNGR peptide for PET imaging of CD13 expression with ovarian cancer xenograft

**DOI:** 10.7150/jca.49628

**Published:** 2021-01-01

**Authors:** Yi Yang, Jun Zhang, Huifeng Zou, Yang Shen, Shengming Deng, Yiwei Wu

**Affiliations:** 1 Department of Nuclear Medicine, the First Affiliated Hospital of Soochow University, Suzhou, Jiangsu 215006, China.; 2 Department of Nuclear Medicine, the Affiliated Suzhou Science & Technology Town Hospital of Nanjing Medical University, Suzhou, Jiangsu 215153, China.; 3 Department of Nuclear Medicine, Taizhou People's Hospital, Taizhou, Jiangsu 225300, China.

**Keywords:** Tumor angiogenesis, NGR peptide, CD13, microPET imaging, ^68^Ga labeling

## Abstract

**Introduction:** Previous studies have shown that peptides containing the asparagine-glycine-arginine (NGR) sequence can specifically bind to CD13 (aminopeptidase N) receptor, a tumor neovascular biomarker that is over-expressed on the surface of angiogenic blood vessels and various tumor cells, and it plays an important role in angiogenesis and tumor progression. In the present study, we aimed to evaluate the efficacy of a gallium-68 (^68^Ga)-labeled dimeric cyclic NGR (cNGR) peptide as a new molecular probe that binds to CD13 *in vitro* and *in vivo*.

**Materials and Methods:** A dimeric cNGR peptide conjugated with 1,4,7,10-tetraazacyclododecane-N,N',N'',N'''-tetraacetic acid (DOTA) was synthesized and labeled with ^68^Ga. *In vitro* uptake and binding analyses of the ^68^Ga- DOTA-c(NGR)_2_ were performed in two ovarian tumor cell lines, ES2 and SKOV3, which had different CD13 expression patterns. An* in vivo* biodistribution study was performed in normal mice, and micro positron emission tomography (PET) imaging was conducted in nude mice bearing ES2 and SKOV3 tumors.

**Results:** ^68^Ga-DOTA-c(NGR)_2_ was prepared with high radiochemical purity (>95%), and it was stable both in saline at room temperature and in bovine serum at 37°C for 3 h. *In vitro* studies showed that the uptake of ^68^Ga-DOTA-c(NGR)_2_ in ES2 cells was higher compared with SKOV3 cells, and such uptake could be blocked by the cold DOTA-c(NGR)_2_. Biodistribution studies demonstrated that ^68^Ga-DOTA-c(NGR)_2_ was rapidly cleared from blood and mainly excreted from the kidney. MicroPET imaging of ES2 tumor xenografts showed the focal uptake of ^68^Ga-DOTA-c(NGR)_2_ in tumors from 1 to 1.5 h post-injection. The high-contrast tumor visualization occurred at 1 h, corresponding to the highest tumor/background ratio of 10.30±0.26. The CD13-specific tumor targeting of the ^68^Ga-DOTA-c(NGR)_2_ was further supported by the reduced uptake of the probe in ES2 tumors by co-injection of the unlabeled cold peptide. In SKOV3 tumor models, the tumor was not obviously visible under the same imaging conditions.

**Conclusions:** ^68^Ga-DOTA-c(NGR)_2_ was easily synthesized, and it showed favorable CD13-specific targeting ability by *in vitro* data and microPET imaging with ovarian cancer xenografts. Collectively, ^68^Ga-DOTA-c(NGR)_2_ might be a potential PET imaging probe for non-invasive evaluation of the CD13 receptor expression in tumors.

## Introduction

Aminopeptidase N, also known as cluster of differentiation 13 (CD13), is a zinc-dependent membrane-bound exopeptidase that is usually up-regulated on the endothelium of tumor neovasculature and in various solid cancers, including melanoma, prostate, ovarian, lung and breast cancer 1-4. CD13 is a key regulator involved in angiogenesis and tumor progression, and it is also a key binding receptor for peptides containing the asparagine-glycine-arginine (NGR) sequence 5-7. In recent years, NGR peptide-based imaging studies have become an intriguing approach in CD13-targeting diagnosis and therapy of cancer.

Ovarian cancer (OVCA), a serious threat to women's health, is characterized by high recurrence rate and high mortality. It is the third most common gynecologic malignant tumor after endometrial carcinoma and cervical cancer in China 8. Many studies have focused on the treatment of OVCA by targeting CD13 4, 9, 10. However, CD13 is differentially expressed in OVCA tissues and cell lines, such as ES2 and SKOV3 cells, due to the heterogeneity of the disease. ES2 cells are intensely positive for CD13, while SKOV3 cells express CD13 at a low level 11.

Radio-labeled NGR peptides have been investigated for CD13 imaging with tumor xenografts 12-15. The cyclic peptides have been found to be more stable than the linear ones because the former is more resistant to enzymolysis 16. In addition, the dimeric NGR peptide shows a higher CD13 affinity than the monomer as the dimer can bind to more sites in target cells 17, 18. In the present study, we designed and synthesized a novel ^68^Ga-labeled dimeric cyclic NGR (cNGR) peptide and investigated its potential for non-invasive positron emission tomography (PET) imaging of CD13 expression with OVCA mouse xenografts.

## Materials and Methods

### General materials

**Figure 1:**
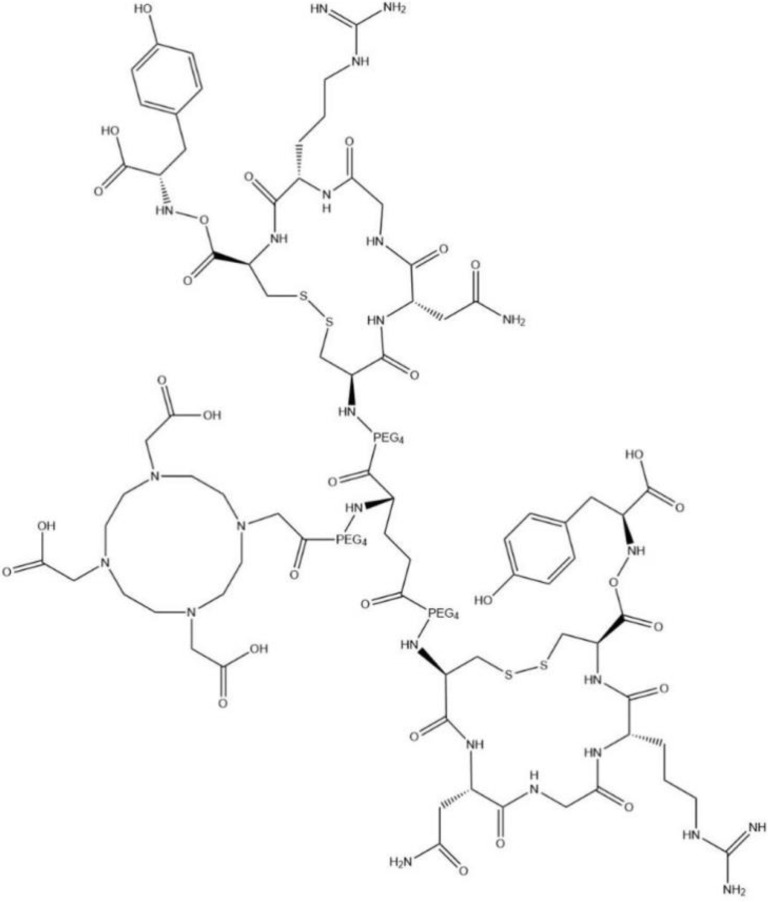
Structural formula of DOTA-c(NGR)_2_; chemical formula: C99H159N25O41S4; molecular weight: 2,483.74.

All chemicals (reagent grade), unless specifically stated, were purchased from commercial suppliers and used without further purification. DOTA-c(NGR)_2_ (**Fig. 1**) was synthesized using standard F-moc solid-phase chemistry by Sangon-Peptide Biotech Co., Ltd. (Ningbo, China) with a purity of 98.X% as analyzed by high-performance liquid chromatography (HPLC) and mass spectrometry (MS). ^68^GaCl_3_ was produced from ^68^Ge-^68^Ga radionuclide generator (ITG GmbH, Oberding, Germany) by elution with 5 mL of 0.05 M HCl.

### Labeling and characterization of ^68^Ga-DOTA-c(NGR)_2_

A DOTA-c(NGR)_2_ stock solution of 5 mg/mL was prepared in deionized water. A volume of 80 μL 1 mol/L HEPES (pH 5.0) was added to 200 μL ^68^GaCl_3_ (37-74 MBq) eluent, and then the mixture was incubated with 40 μL DOTA-c(NGR)_2_ stock solution in a water bath at 95°C for 10 min. Quality control was performed by radio-HPLC (Agilent Technologies, Santa Clara, CA, USA) with a VP-ODS C18 column (Shimadzu, Kyoto, Japan) and an online radioactivity detector (Zonkia Scientific Instruments Co., Ltd., Anhui Province, China). The mobile phase was composed of solvent A, 0.1% trifluoroacetic acid (TFA) in water, and solvent B, 0.1% TFA in acetronitrile. The flow rate was set at 1 mL/min, and the UV wavelength was set at 220 nm. The mobile phase was gradiently changed from 20% solvent B to 30% solvent B in 20 min.

To evaluate the *in vitro* stability, ~3.7 MBq of ^68^Ga-DOTA-c(NGR)_2_ was incubated in phosphate-buffered saline (PBS) at room temperature or bovine serum at 37 °C, respectively. The radiochemical purity was determined at time points of 30 min and 1, 2 and 3 h.

### Cell culture and animal model

The human OVCA ES2 and SKOV3 cells were obtained from Nanjing Keygen Biotech Co., Ltd. (Nanjing, China). ES2 cells were maintained as monolayer cultures in McCoy's medium 5A supplemented with 10% fetal bovine serum (FBS), and SKOV3 cells were cultured in RPMI-1640 medium supplemented with 10% FBS. Both cell lines were incubated at 37°C in a humidified atmosphere containing 5% CO_2_. Cells in log phase were harvested by trypsinization, washed with PBS twice and adjusted to 5×10^7^/mL in PBS. A volume of 0.1 mL single-cell suspension was subcutaneously injected into the front flank of each female BALB/c-neu nude mouse (~4-6 weeks old, body weight of 18-25 g; Slac Laboratory Animal, Shanghai, China). Animal models were used for PET imaging experiments when xenografts grew to 500-1,000 mm^3^. All animal-related studies were approved by the Institutional Animal Care and Use Committee of Soochow University.

### Flow cytometry analysis

ES2 and SKOV3 cells were harvested by trypsinization, washed twice with PBS, and adjusted to a density of 2×10^5^/mL with PBS. The cells were labeled with mouse-anti-human CD13 monoclonal antibody and incubated at 4°C for 30 min, and then the cells were washed with 1 mL cold PBS. Subsequently, the cells were incubated with the secondary antibody labeled with phycoerythrin at 4°C for 30 min. The cells were washed twice and resuspended in 500 μL PBS. Then the labeled cells were analyzed with a flow cytometer (FC-500; Beckman Coulter Inc., Sykesville, MD, USA) to quantify the CD13 expression of OVCA cells.

### Immunohistochemical staining

The xenografted tumors were excised and fixed in 4% buffered formalin (pH 7.0). Sections of paraffin-embedded tumor tissues were baked in an oven at 65°C for 2 h, dewaxed in dimethylbenzene twice, and dehydrated in deionized water and a gradient of alcohol (80%, 95% and 100%). Antigens on the sections were retrieved with antigen repair solution (0.01 M citric acid buffer, pH 6.0), and the endogenous peroxidase activity was eliminated by 3% freshly prepared H_2_O_2_. After blocking in 3% BSA (SW3015; Solarbio, Beijing, China) at 37°C for 30 min, the sections were incubated with adequate diluted rabbit anti-CD13 (1:150; Nanjing Keygen Biotech) in a wet box at 4°C for 12 h, followed by the addition of polymeric horseradish peroxidase-labeled rabbit IgG as the secondary antibody. After incubation at 37°C for 30 min and three washes with PBS, sections were stained with a substrate-chromogen solution containing 0.025% 3, 3'-diaminobenzidine for 10 min and counterstained with hematoxylin for 3 min before microscopic observation at low (200×) and high (400×) magnifications.

### *In vitro* cell binding assay

To study the *in vitro* binding affinity and specificity of ^68^Ga-DOTA-c(NGR)_2_ to CD13, ES2 and SKOV3 cells were seeded into 6-well plates at a density of 1×10^6^ cells/well and cultured overnight. Subsequently, 37 KBq ^68^Ga-DOTA-c(NGR)_2_ was added into each well, followed by incubation at 37°C for 5, 15, 30 min, 1 and 2 h. Then the supernatant was suctioned, and the cells were washed with PBS for three times and harvested with 0.25% trypsin. Both the supernatant and cell suspension were collected and counted using a gamma counter (CRC-55tR; Capintec, Florham Park, NJ, USA).

Cell-based competitive binding assay was performed with ES2 cells. The cells were incubated with 37 KBq ^68^Ga-DOTA-c(NGR)_2_ in the presence of increasing concentrations of unlabeled DOTA-c(NGR)_2_ (0.2-3.2 μg/mL). After 2 h of incubation at 37°C, the cells were collected and measured using the same method as above-mentioned. The experiments were performed in triplicate. The data were fitted with non-linear regression using GraphPad Prism 7.0 (GraphPad Software, San Diego, CA, USA) to obtain the 50% inhibitory concentration (IC_50_).

### Biodistribution studies

Biodistribution studies were performed using 30 normal ICR (Institute of Cancer Research) mice (18-30 g; age, 4 weeks). At 5, 15, 30, 60 min and 2 h post injection of 3.7 MBq/0.1 mL ^68^Ga-DOTA-c(NGR)_2_ via the tail vein, mice (n = 6 per time point) were sacrificed by cervical dislocation. The blood, muscle and other organs of interest (brain, heart, liver, lungs, spleen, pancreas, kidneys, stomach, small intestine and femur) were immediately harvested, weighed and counted with a gamma counter. Data were normalized to the time of injection and expressed as percent injected dose per gram (% ID/g).

### MicroPET imaging and blocking experiments

Two groups of female Balb/c nude mice (n=6/group) bearing ES2 tumors were used to conduct static microPET imaging with an Inveon microPET scanner (Siemens Medical Solutions, Erlangen, Germany). The first group was injected with 7.4 MBq ^68^Ga-DOTA-c(NGR)_2_ via tail vein under anesthesia with 1%-2% isoflurane, and a series of static image data, 10 min for each time point, were collected at 30 min, 1, 1.5, and 2 h post injection. For blocking studies, the second group of mice was injected with 100 μg unlabeled peptide in 100 μL saline via tail vein, immediately followed by administration of ^68^Ga-DOTA-c(NGR)_2_, and then images were acquired at 30 min, 1, 1.5 and 2 h post-injection, each for 10 min. The obtained images were processed using Siemens Inveon Research Workplace 4.0 (IRW 4.0) and reconstructed using three-dimensional ordered-subset expectation maximization. Regions of interest (ROIs) were drawn over the tumor (T) and muscle of the contralateral forelimb, and the latter served as the background (B). Moreover, %ID/g of tumors and the T/B ratio were determined. The same studies were repeated in female Balb/c nude mice bearing SKOV3 tumors without the blocking experiments.

### Statistical analysis

All quantitative data were presented as the mean ± standard deviation (SD). Statistical analysis was conducted by one-way analysis of variance and Student's *t*-test. *P* < 0.05 was considered statistically significant.

## Results

### Radiochemistry and stability

**Figure 2:**
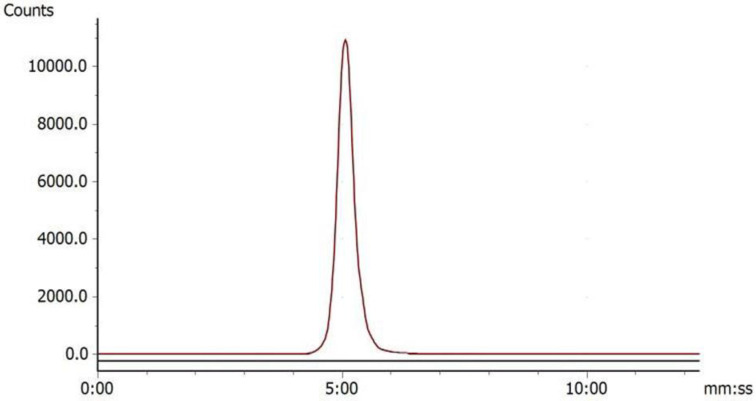
Radio-HPLC of ^68^Ga-DOTA-c(NGR)_2_ (retention time was 5.23 min).

**Figure 3:**
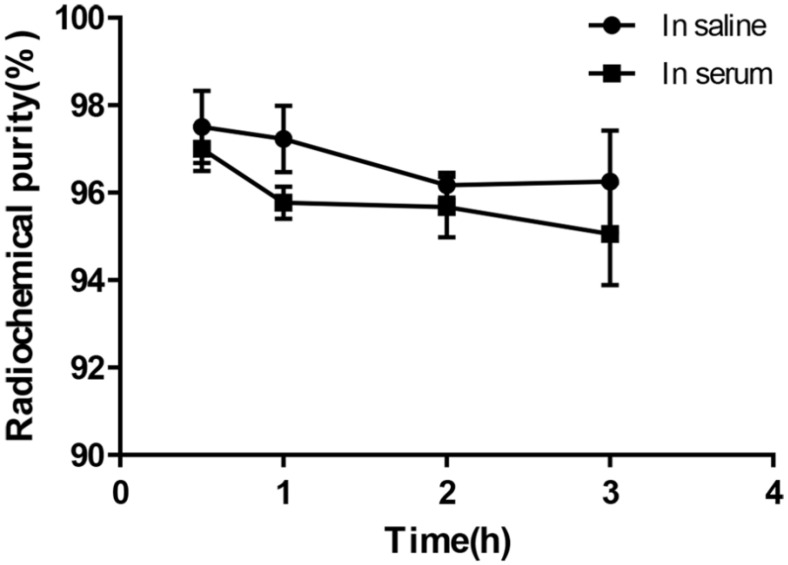
*In vitro* stability of ^68^Ga-DOTA-c(NGR)_2_ in saline at room temperature and in bovine serum at 37 °C for 30 min, 1 h, 2 h and 3 h.

Under the condition of bathing at 95°C for 10 min, ^68^Ga-DOTA-c(NGR)_2_ was easily prepared with a high labeling rate of 98.01% ± 1.44%, and further purification was not necessary. The retention time of ^68^Ga-DOTA-c(NGR)_2_ on radio-HPLC was 4.86 ± 0.27 min (**Fig. 2**). For the stability study, the radiochemical purity was >96% in saline and >95% in bovine serum after 3 h of incubation (**Fig. 3**).

### Fluorescence-activated cell sorting (FACS) and immunohistochemical staining

**Figure 4:**
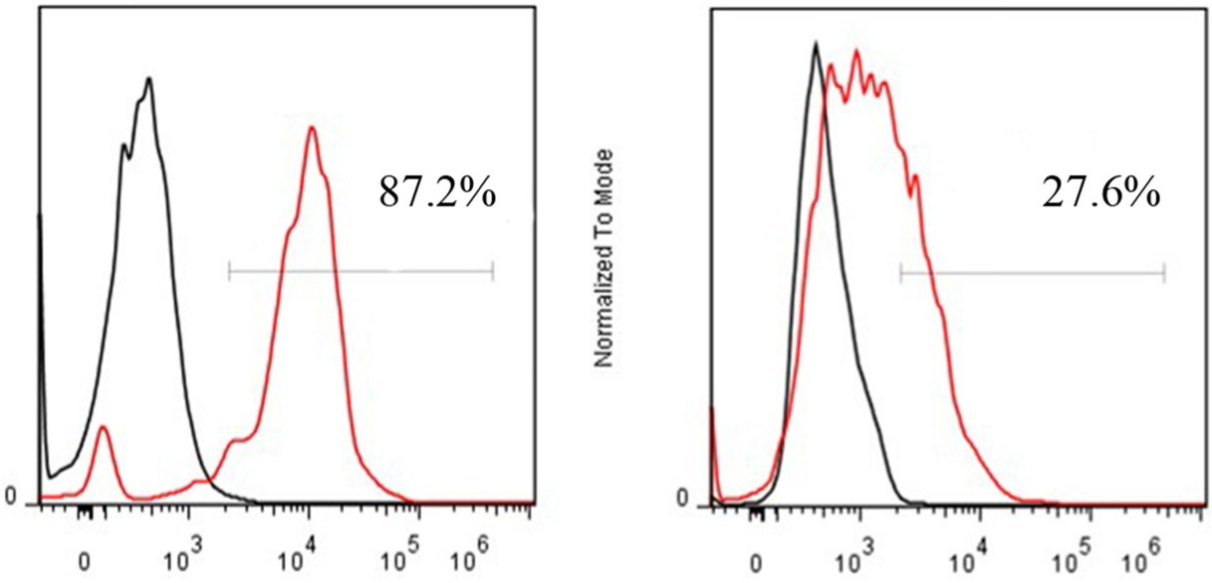
FACS of CD13 expression on ES2 (A) and SKOV3 (B) cells.

**Figure 5:**
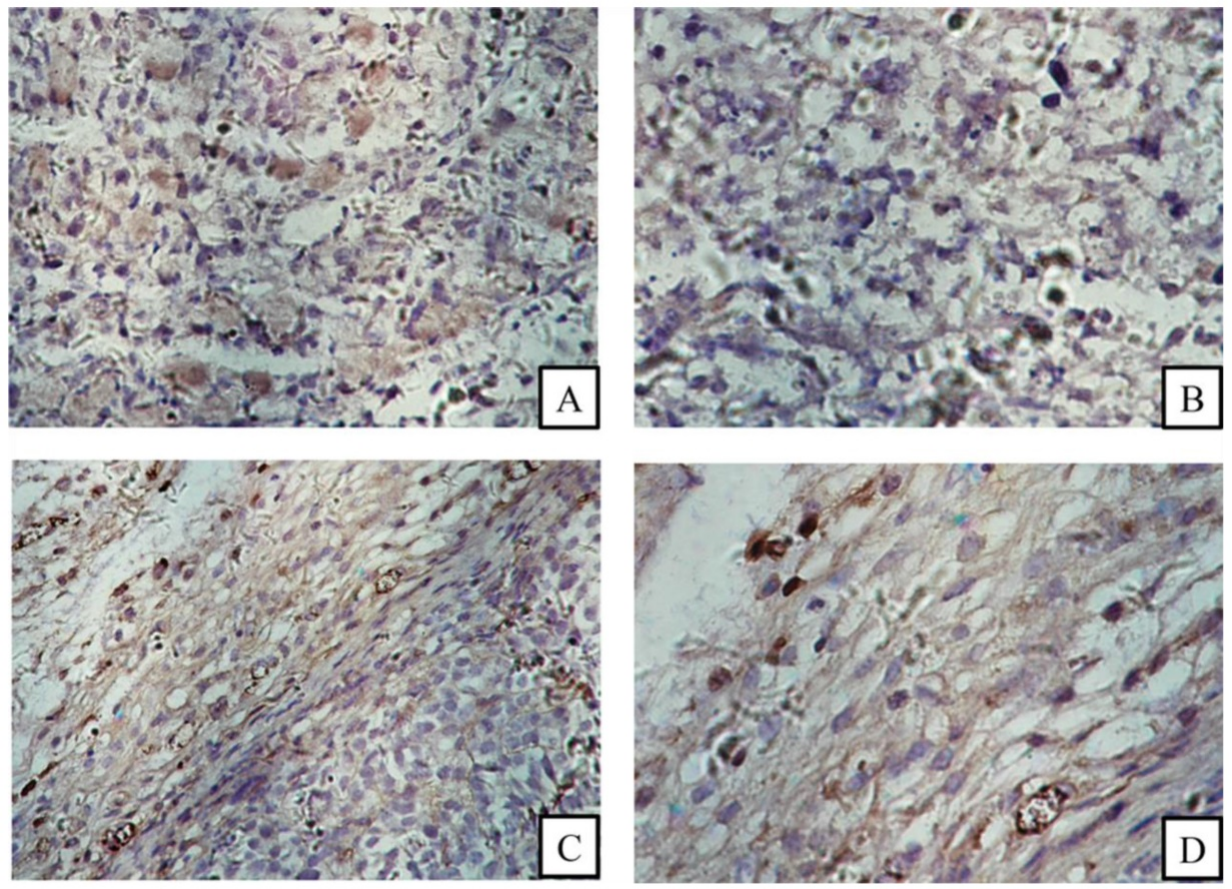
Immunohistochemical staining. (A) and (B) CD13 expression in SKOV3 tumor tissue sections; (C) and (D) CD13 expression in ES2 tumor cells (black arrows) and tumor vascular endothelia (white arrows). (A and C magnification × 200, B and D magnification ×400).

FACS revealed that the CD13 expression rates of ES2 cells and SKOV3 cells were 87.2% and 27.6%, respectively (**Fig. 4**). In accordance with the results of FACS, immunohistochemical staining showed that CD13 was significantly expressed on the membranes of the tumor and endothelial cells of the new vasculature in ES2 tumor tissue, and its expression in ES2 tumor tissue was significantly higher compared with the SKOV3 tumor tissue (**Fig. 5**).

### *In vitro* cell-binding assay

**Figure 6:**
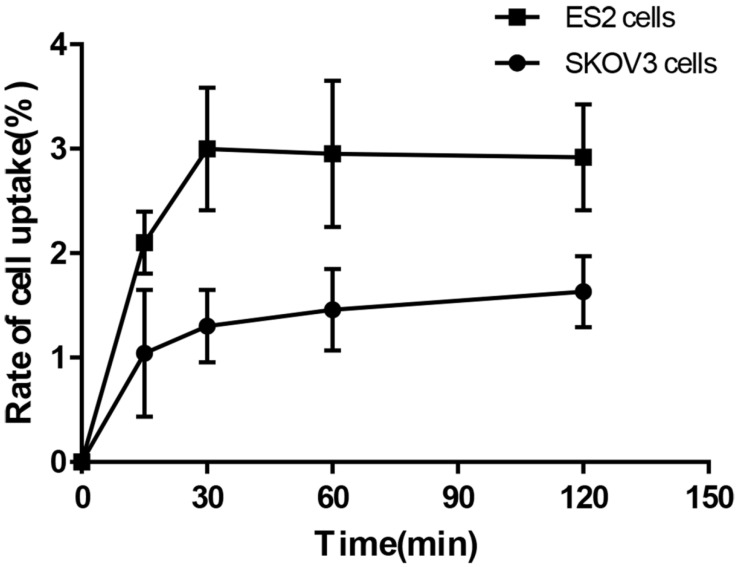
Uptake of ^68^Ga-DOTA-c(NGR)_2_ in ES2 and SKOV3 cells.

**Figure 7:**
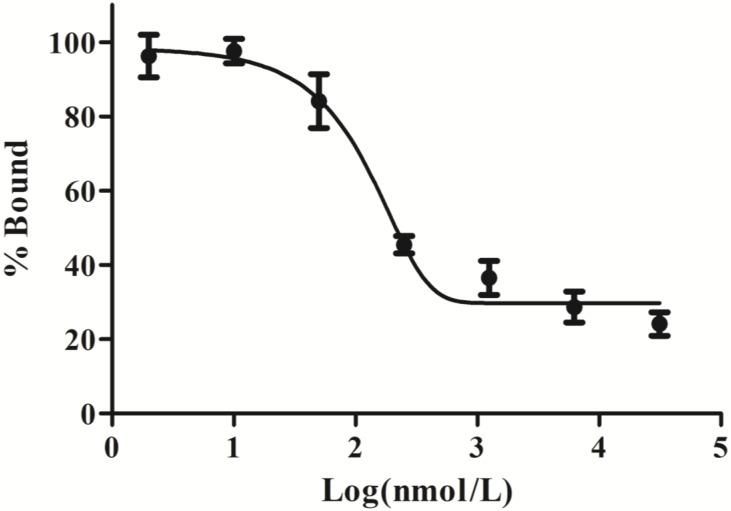
Competitive binding assay. The uptake in ES2 cells could be competitively inhibited by unlabeled DOTA-c(NGR)_2_ in a dose-dependent manner, and the IC50 value was 160.1 nM.

Cell-binding studies were performed with ES2 cells and SKOV3 cells, and blocking studies were performed with ES2 cells. Significant binding differences were observed between ES2 cells and SKOV3 cells (**Fig. 6**). The binding of ^68^Ga-DOTA-c(NGR)_2_ to ES2 cells was rapid and almost saturated within 30 min, and the highest binding of 3.00% ± 0.59% was achieved after 2 h of incubation, which was significantly higher compared with SKOV3 cells at all time points with a highest binding of 1.79% ± 0.34% (*P*<0.05) (Fig. 6). Cell-based competitive binding assay demonstrated that the binding of ^68^Ga-DOTA-c(NGR)_2_ to ES2 cells was blocked by DOTA-c(NGR)_2_ in a dose-dependent manner, and the calculated IC_50_ value was 160.1 nM (**Fig. 7**).

### Biodistribution studies

**Figure 8:**
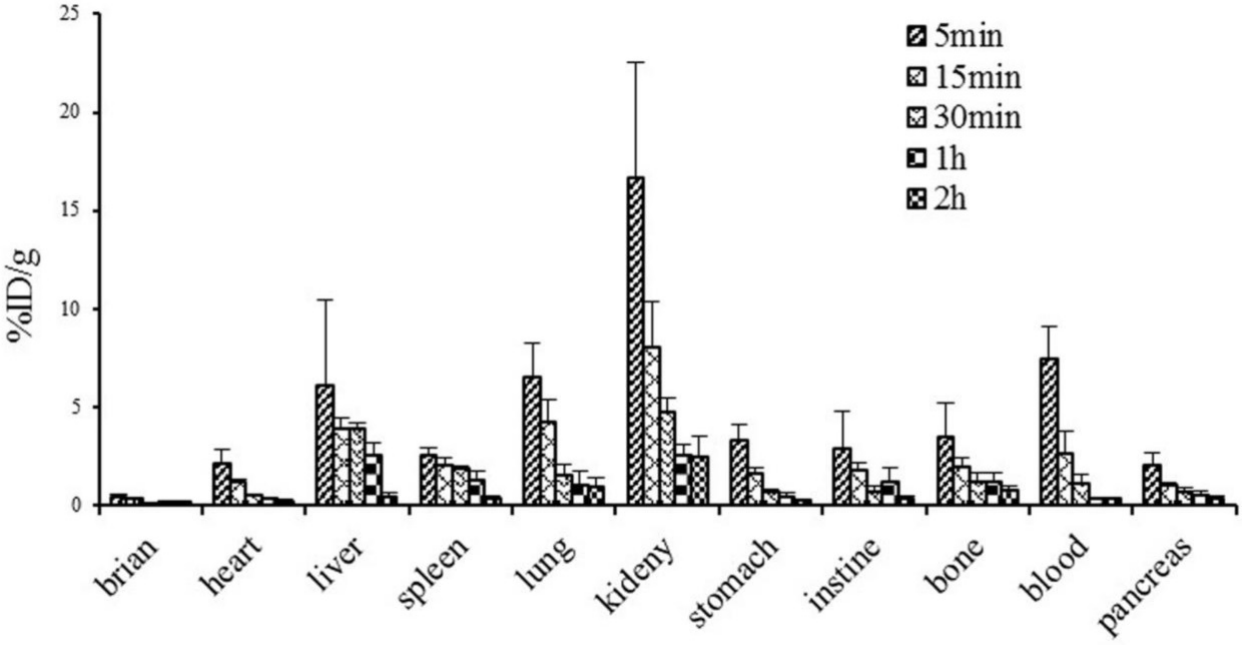
Biodistribution of ^68^Ga-DOTA-c(NGR)_2_ in normal mice (n=6).

**Figure 8** illustrates the dynamic biodistribution results of ^68^Ga-DOTA-c(NGR)_2_ in healthy mice. The highest uptake of the tracer was found in the kidney, which peaked at 5 min (16.7±5.80% ID/g) and then was rapidly decreased by more than 50% at 30 min and 85% at 120 min post-injection. Liver and lung were two other organs with relatively high uptake of the tracer. Quick clearance of ^68^Ga-DOTA-c(NGR)_2_ from blood was observed, and the blood uptake was 7.44 ± 1.64% ID/g at 5 min and 0.31 ± 0.05% ID/g at 60 min post-injection. Activities in brain, heart, stomach, intestines, spleen, pancreas and bone were at lower levels.

### MicroPET imaging and blocking experiments

**Figure 9:**
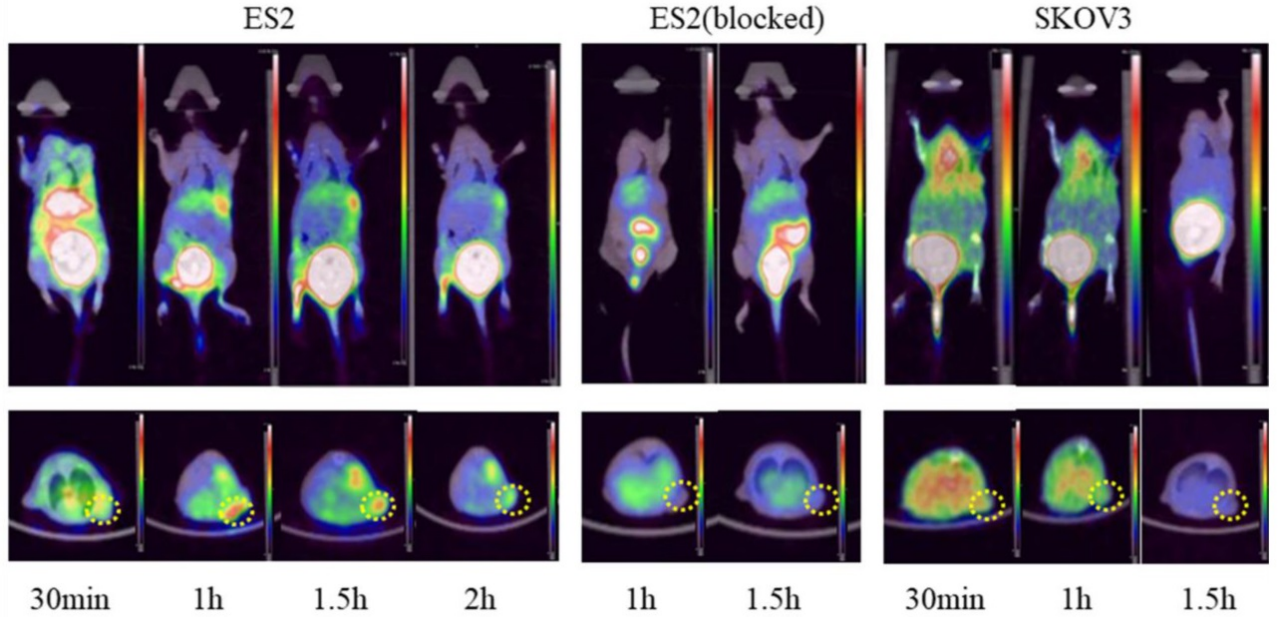
Representative micro-PET images of ^68^Ga-DOTA-c(NGR)_2_ in xenograft models. Dotted circles indicate the tumor regions.

Post-intravenous injection with ^68^Ga-DOTA-c(NGR)_2_, a series of microPET images of ES2 tumor-bearing mice were collected at 30 min, 1 and 1.5 h and represented by coronal and transverse views (**Fig. 9**). Significant accumulation of radioactivity in the bladder was observed at all time points. High focal accumulation in tumor was visualized at 1 and 1.5 h, and then it was reduced at 2 h. Quantitative analysis with ROI revealed that the uptake of ^68^Ga-DOTA-c(NGR)_2_ in ES2 tumors was 0.62 ± 0.09% ID/g at 1 h and 0.53 ± 0.08% ID/g at 1.5 h, whereas the uptake in SKOV3 tumors at the same time points was 0.32 ± 0.03% ID/g and 0.24 ± 0.05% ID/g, respectively. The T/B ratios in ES2 tumors were 10.30 ± 0.26 at 1 h and 8.04±1.75 at 1.5 h, while such ratios became only 3.99 ± 0.18 at 1 h and 4.24 ± 0.73 at 1.5 h in SKOV3 tumors. The uptake in ES2 tumors was only 0.16 ± 0.03% ID/g at 1 h and 0.14 ± 0.02% ID/g at 1.5 h in the blocking experiment, indicating that the specific targeting of ^68^Ga-DOTA-c(NGR)_2_ to the tumor was significantly blocked by the cold peptide.

## Discussion

The effective inhibition of tumor angiogenesis may arrest tumor progression and has shown synergistic effect in other cancer treatments 19, 20. As one targeted tumor therapy, anti-angiogenic regimen can actually benefit only a few patients due to the biological heterogeneity of tumors 21, 22. Molecular imaging using radio-labeled tumor-targeting probes can be used to select patients, dynamically assess response to therapy, and therefore predict efficacy of treatment 23. ^68^Ga, a generator-based positron radioisotope, is relatively easy to obtain by ^68^Ge/^68^Ga radionuclide generators, and it shows high-quality images on PET 24. CD13 is one of attractive targets of tumorigenic angiogenesis for nuclear imaging. In the present study, we investigated a ^68^Ga-labeled dimeric cNGR linked by PEG-4 for CD13 specific-targeting PET imaging. Like some previous studies, we employed ES2 cells, which were intensely positive for CD13, and SKOV3 cells, which expressed a low level of CD13, to ensure that the efficacy of ^68^Ga-DOTA-c(NGR)_2_ as a new molecular probe that binds to CD13-positive OVCA cells was comparable 9, 10, 25.

^68^Ga-DOTA-c(NGR)_2_ with high radiochemical purity was obtained via relatively simple labeling steps without further purification, and the yield remained stable in bovine serum after 3 h. The specificity and cellular binding kinetics of the probe were verified by *in vitro* experiments with two OVCA cell lines. The potential of ^68^Ga-DOTA-c(NGR)_2_ for PET imaging of tumor CD13 expression was also demonstrated by the *in vivo* experiments with the OVCA model.

In our previous study, ^68^Ga-labeled linear NGR conjugated with DOTA, ^68^Ga-DOTA-NGR, has been synthesized, which shows high selectivity and affinity for CD13 and significant uptake in CD13-positive lung tumors 12. However, only a few studies have investigated radio-labeled NGR in OVCA. Faintuch et al. have evaluated the uptake of cNGRyk peptide radio-labeled with ^99m^Tc in OVCA cell namely OVCAR-3 and found that the *in vivo* tumor uptake is pronounced 26. Meng et al. have synthesized NGR peptide-conjugated Cy5.5 labeled iron oxide (Fe_3_O_4_-Cy5.5-NGR) nanoparticles as a targeted NIRF/MR dual-modal imaging nanoprobe and indicated that CD13 can be utilized as a suitable target for OVCA specific imaging 27. In line with these studies, we observed that the new ^68^Ga-labeled dimeric cNGR was characterized as a promising diagnostic candidate for OVCA.

In recent years, many inhibitors of APN/CD13 have been designed and synthesized to treat OVCA via reducing the activity and expression of APN/CD13 9, 10, 28. However, no relationship has been demonstrated between the expression of CD13 in OVCA and clinical or pathologic variables of the patients 29. Therefore, the CD13 expression in tumors should be evaluated through biopsy before treatment. However, there are associated risks and contraindications of the biopsy procedure. Moreover, the result is complicated by the heterogeneous spatial expression of CD13 within a tumor. Molecular imaging using radio-labeled peptides can provide a non-invasive means to detect the expressions of special receptors throughout an entire tumor simultaneously, without the need for histological examination 30, 31. In the present study, a higher uptake of radiotracer was found in ES2 cells compared with SKOV3 cells, indicating that ^68^Ga-DOTA-c(NGR)_2_ could be used to evaluate the CD13 expression in OVCA.

It is worth noting that our work is only a preliminary study on the physicochemical and biological characteristics of a newly designed cyclic dimeric probe based on the NGR sequence. It was not compared with other probes of NGR derivatives in previous studies under the same experimental conditions.

## Conclusions

A new ^68^Ga-labeled dimeric cNGR, ^68^Ga-DOTA-c(NGR)_2_, was easily synthesized with high radiochemical purity and stability. Both *in vitro* and *in vivo* data demonstrated the high selectivity and affinity of ^68^Ga-DOTA-c(NGR)_2_ for CD13. Collectively, ^68^Ga-DOTA-c(NGR)_2_ might be a potential PET imaging probe for evaluating the CD13 expression in tumor.

## Abbreviations

^68^Ga: gallium-68
NGR: asparagine-glycine-arginine
cNGR: cyclic NGR
cNGR2: cyclic NGR dimer
DOTA: 1,4,7,10-tetraazacyclododecane-N,N',N'',N'''-tetraacetic acid
PET: Positron emission tomography
CD13: cluster of differentiation 13
OVCA: ovarian cancer
FBS: fetal bovine serum
% ID/g: percent injected dose per gram
ROI: region of interest
FACS: fluorescence-activated cell sorting
